# Intussusception of the bowel in a young woman: A case report

**DOI:** 10.1002/ccr3.6309

**Published:** 2022-09-16

**Authors:** Peyman Hejazi, Saeed Yousefi, Hossein Hemmati, Niloofar Faraji, Fatemeh Mohammadyari

**Affiliations:** ^1^ Razi Clinical Research Development Unit, Razi Hospital Guilan University of Medical Rasht Iran; ^2^ Department of General Surgery, School of Medicine Road Trauma Research Center, Razi Hospital Guilan University of Medical Sciences Rasht Iran; ^3^ School of Medicine Guilan University of Medical Sciences Rasht Iran

**Keywords:** adenocarcinoma, intussusception, Meckel's diverticulum, young woman

## Abstract

Intussusception is a condition in which a segment of the gastrointestinal tract invaginates into the lumen of another segment. Adult intussusception is less common than juvenile intussusception in terms of cause, appearance, and treatment. Because the clinical picture can be quite atypical and difficult to interpret, it is frequently misdiagnosed at first. Herein, we report the case of a previously healthy 23‐year‐old female patient who presented to the Emergency Department (ED) with acute abdominal pain, vomiting, and diarrhea for 1 day following her last menstrual period. Ileocecal intussusception was discovered throughout the investigation. She was rushed for open abdominal surgery. Meckel's diverticulum was found as a pathologic lead point in the resected specimen, with no evidence of malignancy. Although intussusception is rare in adults, it should be considered in patients who have nonspecific stomach pain.

## Graphical abstract

Intussuception in adults is a rare cause of intestinal obstruction. Early imaging like ultrasonography and CT scan can lead us to make the diagnosis soon. Laparotomy is the choice of surgery for small bowel obstruction. The extension of resection depends on the pathologic cause of intussusception as the lead point.

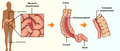

## 1 BACKGROUND

Paul Barbette first characterized intussusception as a telescope‐like invagination of the proximal segment of the intestine (intussusceptum) into the distal portion of the intestine (intussuscipiens).^1^ Prolapse of a proximal intestinal segment into a distal intestine segment is now the accepted definition.^2^ Any abnormality in the intestine that alters the usual pattern of peristalsis raises the risk of intussusception.^3^ The majority of patients present with a clinical presentation of intestinal obstruction.^4^ Adult intussusception is far less prevalent, accounting for just 5% of all intussusceptions, 1% of all intestinal obstructions, 0.08% of all abdominal surgery, and 0.003%–0.02% of all hospital admissions. Intussusception is expected to affect 2–3 cases per 100,000 people each year in adults. In 90% of cases, idiopathic intussusception in children is benign and can be safely minimized.^5^ Adult intussusception, on the other hand, differs from pediatric intussusception in several ways. Nearly 90% of adult intussusception is caused by a pathologic disease that acts as a lead point, such as polyps, carcinomas, Meckel's diverticulum, and benign neoplasms, which are frequently identified intraoperatively.^2^ Chronic marijuana usage is also a less common and more recent probable cause of adult intussusception.^6^ Also, patients with acquired immune deficiency syndrome (AIDS) had a higher prevalence of intussusception, according to reports.^7^, ^8^ Because of the ambiguous and intermittent character of symptoms in adults, diagnosis is frequently delayed.^9^ The most sensitive diagnostic approach is computed tomography (CT), which can identify intussusceptions with and without a lead point.^2^ Adult intussusceptions require an exploratory laparotomy for surgical treatment, as opposed to children's intussusceptions, which are treated nonoperatively.^10^ An adult female with ileocecal intussusception caused by Meckel's diverticulum is described in this article.

## 2 CASE PRESENTATION

**FIGURE 1:**
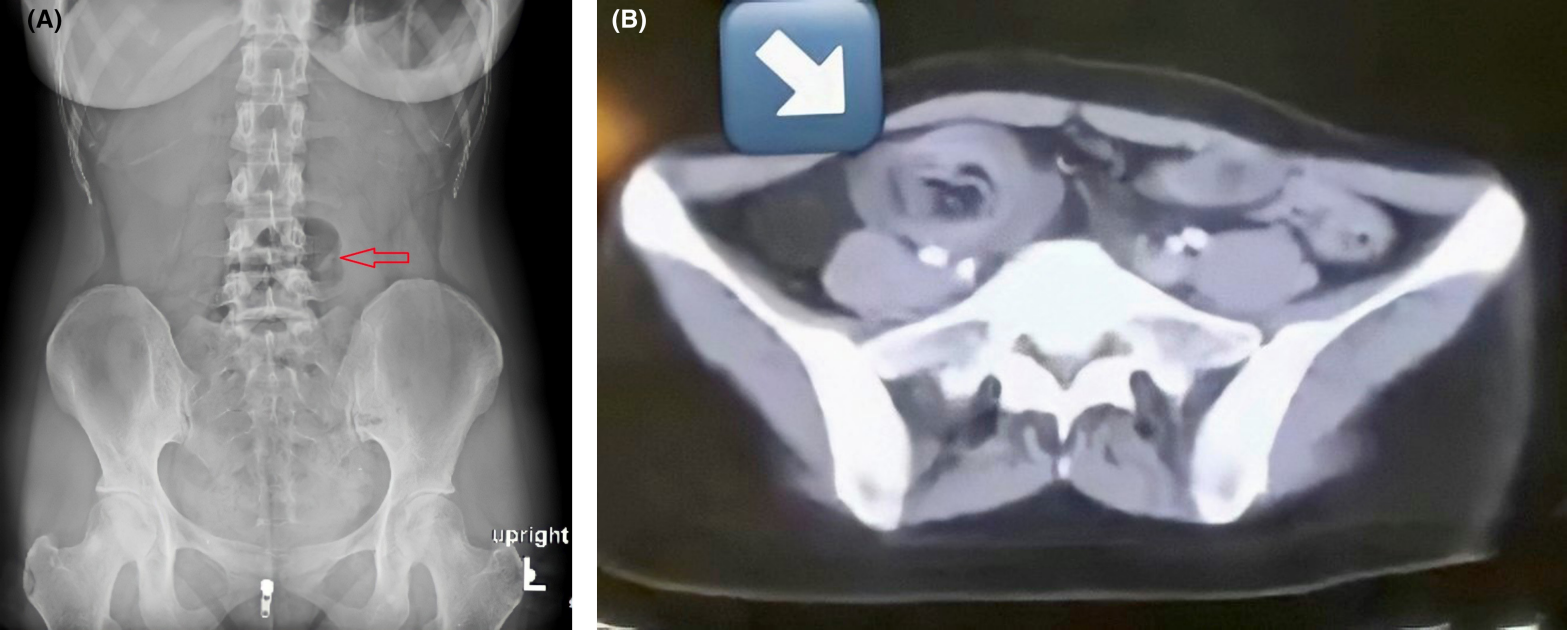
(A) Plain abdominal radiography suggests intestinal obstruction (arrow). (B) CT scan of the abdomen; Target sign appearance at the right side of the abdomen (arrow)

**FIGURE 2:**
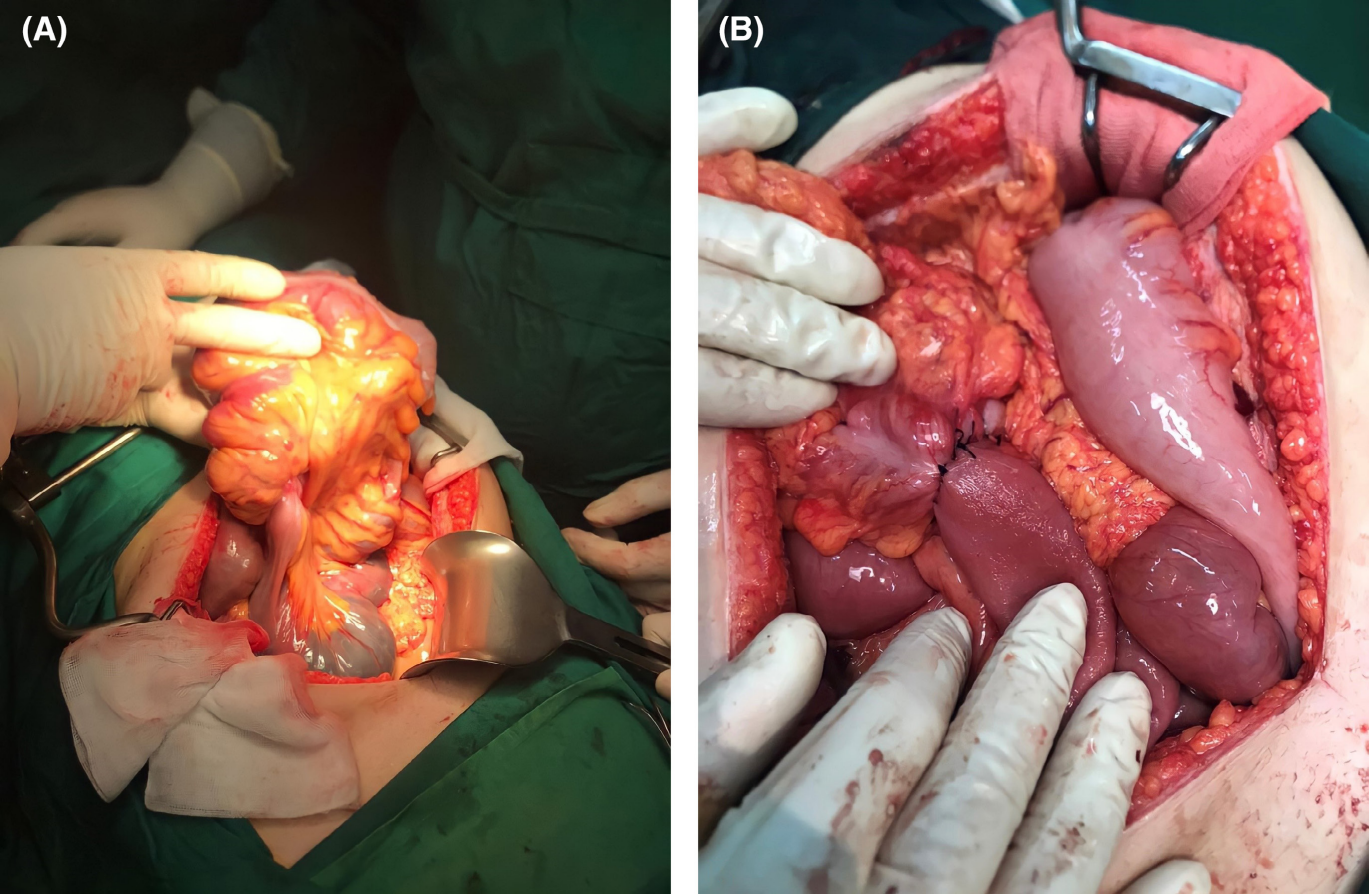
(A) Ileocecal intussusception. (B) End‐to‐side anastomosis between the ileum and the ascending colon

This is a case of a previously healthy 23‐year‐old woman who was admitted as an emergency with intermittent colicky abdominal pain, vomiting, and diarrhea for 1 day following her last menstrual period. Her vital signs were unremarkable, but a physical examination revealed generalized abdominal tenderness, which over time turned into guarding and rebound tenderness in the course of hospitalization. There is no organomegaly and also no masses were palpable. Her lab tests came back normal. A doughnut sign was visible on transabdominal ultrasound, as well as slight distension of intestinal loops in the pelvic cavity. In the retroperitoneal space, no apparent pathology or lymphadenopathy could be found. An abdominal X‐ray revealed closed loops, indicating intestinal obstruction (Figure 1A). She underwent abdominal contrast‐enhanced computed tomography (CT), which revealed a thickened ileum with a target appearance, consistent with an ileocecal intussusception (Figure 1B). The choice was taken to correct the intussusception through open abdominal surgery. The patient was placed in the supine position in the operating room after general anesthesia. A laparotomy was performed in the midline. Ileocecal intussusception with mucosal infarction in the cecum was among the surgical findings (Figure 2A). The procedure included an ileocecectomy and a segmental colectomy with no manual reduction. No obvious tumors or other abnormalities suggestive of malignancy were seen during an examination of the abdominal cavity and peritoneum. The ileum and the ascending colon were joined together by a functioning end‐to‐side anastomosis (Figure 2B). According to the pathology report, the diagnosis was Meckel's diverticulum with ectopic gastric mucosa, with no sign of cancer. She had a smooth surgery recovery and was discharged 6 days later.

## 3 DISCUSSION AND CONCLUSION

Intestinal intussusception is a rare disease in the adult population and is more likely to occur in children, which causes 1%–5% of mechanical bowel obstructions.^11^ Intussusception in adults is often brought on by a pathologic lead point in the gut, which is cancerous in up to 77% of cases,^1^, ^12^ although our patient had a benign pathology known as a Meckel's diverticulum with ectopic gastric mucosa, with no sign of cancer. Meckel diverticulum is the most prevalent among numerous lead sites with neoplastic, structural, inflammatory, or vascular/hematological characteristics.^13^ Intussusception can be categorized by location (colocolic, ileocolic, and enteroenteric), or by etiology (idiopathic, benign, or malignant lesions). Additionally, intussusception of the jejunum and stomach in individuals who have had gastrojejunostomies can also be categorized as antegrade or retrograde.^14^ Small intestinal intussusception is mainly caused by lesions inside or outside the intestine, including adenomatous polyps, lipomas, stromal tumors, lymphoma, inflammatory bowel disease, Meckel's diverticulum, postoperative intestinal adhesions, adenocarcinoma, and metastatic carcinoma. Endometriosis, angiolipoma, myoepithelial hamartoma, and appendix mucinous cystadenoma also occur, which have only been reported in case reports.^15^, ^16^, ^17^ Diagnosis of intussusception may be made by imaging techniques. In this case, after a systematic preview, ultrasound sonography demonstrated a pseudo kidney phenomenon and then an abdominal Intravenous and Oral contrast CT scan revealed a thickened ileum with a target sign appearance helped us to reach the final diagnosis.^18^ A “target sign” hypo/hyperdense layers alternate, pointing to intussusception may be visible on the sagittal view, however, on the axial or coronal view, the intussusception will appear as a sausage‐shaped mass.^14^ Most surgeons agree that adult intussusception requires surgical intervention. With all of these, during the COVID‐19 pandemic, surgical specialties will face tremendous challenges, with long‐term effects.^19^, ^20^ Intussusception, an urgent condition of the acute abdomen, necessitates different approaches in children and adults. While in children we can try two times to correct intussusception by contrast or air enema, adults may need to consider surgical intervention. Surgery should not be delayed at all. In our perspective and based on prior studies, the lead point of intussusception will decide the surgical method, and this aberrant state is the main point in the surgical treatment approach. After assessing the pathological cause of the issue, the surgeon should decide whether to advance the procedure as a cancer surgery in the event of any malignant manifestation in the operating room such as a right hemicolectomy in the case of ileocecal intussusceptions in the event of any lymphadenopathy or any pathological evidence of malignancy as the lead point and even seeking assistance from the frozen section and pathology report would be useful. On the other hand, if the lead point was in the background of a benign issue, as a Meckel diverticulum in our case, a segmental colectomy with resection of the lead point would be considered instead of more radical surgery. In addition to giving adequate exposure to surgeons finding any signs of malignancy on the whole level of the abdomen, laparotomy is the choice of surgery in acute intestinal obstruction. Although the laparoscopic method is becoming more and more well‐liked as minimally invasive surgery, there is no consensus on the appropriate setting for laparoscopic therapy for small bowel obstruction.^21^, ^22^

In conclusion, however, intussusception is a rare cause of abdominal pain in adults, it is a diagnosis to consider in patients who have nonspecific abdominal pain because it can be a sign of cancer. In all situations where suspicion exists, emergency physicians should seek early surgical consultation. Diagnosis could be taken by appropriate imaging like ultrasound sonography or an IV/Oral CT scan. The best way to treat adult intussusception is to consider the factors like the frequency of an underlying disease, the anatomic location and extent of intussusception, the evidence of malignancy, and the presence of any associated inflammation, edema, or bowel ischemia. After all, the pathogenic component has a significant impact on surgical selection. The surgeon should choose the best strategy to accomplish it for a multidisciplinary approach during the surgery. As laparotomy is the choice of surgery in acute intestinal obstruction, the decision to consider a radical resection or correcting the intussusception by segmental resection of the involving complex has to be taken due to the pathological reason of the lead point.

## AUTHOR CONTRIBUTIONS

PH, SY performed the surgery. FM wrote the manuscript. NF, HH, and PH revised and edited the case report. FM made the final review of the manuscript before submission. FM was responsible for the conception of the idea, supervision, and editing of the project. All authors read and approved the final manuscript.

## CONFLICT OF INTEREST

The authors declare that they have no competing interests.

## ETHICAL APPROVAL

The study is exempt from ethical approval in our institution.

## CONSENT

Written informed consent was obtained from the patient's legal guardian for publication of this case report and any accompanying images. A copy of the written consent is available for review by the Editor‐in‐Chief of this journal.

## Data Availability

Data sharing is not applicable to this article as no datasets were generated or analyzed during the current study.
